# Peptide Receptor Radionuclide Therapy versus Capecitabine/Temozolomide for the Treatment of Metastatic Pancreatic Neuroendocrine Tumors

**DOI:** 10.3390/cancers16172993

**Published:** 2024-08-28

**Authors:** Rushabh Gujarathi, Joseph Tobias, Sara Abou Azar, Xavier M. Keutgen, Chih-Yi Liao

**Affiliations:** 1 Section of Hematology and Oncology, Department of Medicine, University of Chicago, Chicago, IL 60637, USA; 2 Section of Endocrine Surgery, Department of Surgery, University of Chicago, Chicago, IL 60637, USA

**Keywords:** pancreatic NET, PRRT, CAPTEM, treatment sequencing

## Abstract

**Simple Summary:**

Peptide Receptor Radionuclide Therapy (PRRT) and Capecitabine/Temozolomide (CAPTEM) are cornerstones of systemic therapy for metastatic pancreatic neuroendocrine tumors (PNETs). In clinical practice, most patients receive more than one regimen, and the optimal sequence of treatments for these patients remains poorly understood. We present a real-world comparison of progression-free survival (PFS) between PRRT and CAPTEM in PNETs. Our analysis found that clinical outcomes were comparable to previously reported data in the real-world setting and were similar for both regimens. PFS for patients without extrahepatic metastases and those with MEN1, DAXX, and/or ATRX mutations might be prolonged with PRRT. Patients with grade 3 disease might fare better with CAPTEM initially. Candidates for surgical debulking or those with tumor-induced symptoms may benefit from initial treatment with CAPTEM due to the shorter time to response.

**Abstract:**

**Background:** Peptide Receptor Radionuclide Therapy (PRRT), a form of Radioligand Therapy (RLT), and Capecitabine/Temozolomide (CAPTEM) are cornerstones of systemic therapy for metastatic pancreatic neuroendocrine tumors (PNETs). Data regarding comparative efficacy are lacking. Herein, we compare the efficacy of PRRT vs. CAPTEM as second-line/beyond regimens and treatment sequencing. **Methods:** Clinicopathologic, radiographic, and genomic data were captured for metastatic PNETs seen in our multi-disciplinary NET clinic between 2013 and 2023. The primary outcome was progression-free survival (PFS) after progression on a previous line of systemic therapy. The secondary outcomes were objective response rate (ORR), time to response (TTR), and overall survival (OS). **Results:** Fifty-nine cases were included. PFS was similar in the PRRT (*n* = 29) and CAPTEM (*n* = 30) groups (PRRT = 21.90 months vs. CAPTEM = 20.03 months; HR 0.99; *p* = 0.97). On subgroup analysis, PRRT had longer PFS in cases without extrahepatic metastases (26.47 months vs. 17.67 months; *p* = 0.03) and cases with a mutation in the MEN1, DAXX, and/or ATRX genes (28.43 months vs. 18.67 months; *p* = 0.03). PRRT had reduced PFS in patients with grade 3 disease (7.83 months vs. 16.33 months; *p* = 0.02). ORR did not vary significantly (34.78% vs. 40.91%; *p* = 0.67). CAPTEM responders showed shorter TTR (6.03 months vs. 11.15 months; *p* = 0.03). In patients who received both, OS did not vary based on the sequence (HR 1.20; *p* = 0.75). **Conclusions:** PFS, ORR, and OS are similar when using PRRT vs. CAPTEM as second-line-and-beyond therapy for patients with metastatic PNETs. However, patients with *MEN1*, *DAXX*, and/or *ATRX* mutations or without extrahepatic metastases might better benefit from PRRT and patients with grade 3 disease from CAPTEM. Candidates for surgical debulking or with tumor-induced symptoms may benefit from initial treatment with CAPTEM due to shorter TTR.

## 1. Introduction

Pancreatic neuroendocrine tumors (PNETs) are rare pancreatic neoplasms with an age-adjusted incidence of up to 1 per 100,000 people in the United States [1]. The incidence of PNETs has increased in recent years [2]. Recent estimates indicate that the median 5-year overall survival for patients with metastatic PNETs remains poor at 28% [2].

Most patients with PNETs are diagnosed with advanced or metastatic disease at the time of initial presentation [3]. Treatment options for advanced PNETs include surgical debulking, liver-directed therapies, and systemic therapies [4]. Systemic therapies commonly utilized in PNETs include somatostatin analogs (SSAs), Peptide Receptor Radionuclide Therapy (PRRT), Capecitabine/Temozolomide (CAPTEM), and targeted therapies (everolimus or sunitinib) [4,5]. In clinical practice, most patients receive more than one regimen during the course of their disease, and the sequence of these treatments varies according to patients and physicians [5]. Robust data regarding the optimal sequence of treatments for advanced PNETs are lacking, and results from ongoing prospective clinical trials are eagerly awaited to further inform treatment sequencing [5,6].

Following disease progression on first-line SSA (long-acting octreotide analogs), PRRT and CAPTEM are two subsequent systemic therapies that are commonly used for second-line-and-beyond treatment in advanced PNETs. PRRT, a form of Radioligand Therapy (RLT), consists of radiolabeled somatostatin analogs, which are effective in the management of metastatic gastroenteropancreatic neuroendocrine tumors (GEP-NETs) as most of these tumors express somatostatin receptors [7,8]. The most widely utilized form of PRRT, ^177^Lu-DOTATATE, was approved for use in GEP-NETs based on significant progression-free survival (PFS) benefits reported from the phase III NETTER-1 trial and other prospective studies [9,10]. A meta-analysis of 473 patients treated with ^177^Lu-labelled PRRT reported objective response rates (ORRs) between 17.6% and 43.8% based on RECIST criteria [11]. Although PNETs were not included in NETTER-1, recent data from a large Spanish registry that included 182 PNETs has shown a median PFS of 19.8 months with ^177^Lu-DOTATATE in PNETs [12]. The recently reported primary results from the NETTER-2 study, which included PNETs, demonstrated clinically meaningful efficacy for PRRT in patients with grade 2 and grade 3 well-differentiated gastroenteropancreatic neuroendocrine tumors. Notably, there was a significant improvement seen in PFS among patients who received PRRT (median PFS = 22.8 months vs. 8.5 months with placebo), and the PFS benefit remained significant in the PNET subgroup (HR = 0.34) [13]. CAPTEM is a combination of capecitabine, an oral prodrug that is converted to the antimetabolite 5- fluorouracil (FU), and the oral alkylating agent temozolomide [14,15]. CAPTEM is another cornerstone of systemic therapy for metastatic PNETs, with recent results from the ECOG-ACRIN E2211 study reporting a significant improvement in PFS compared with temozolomide alone. A median PFS of 23.2 months, along with an ORR of 39.7%, was reported in the CAPTEM arm of this study [16]. Although both PRRT and CAPTEM are effective treatment regimens, the optimal sequence of these regimens for metastatic PNET patients is poorly understood [6].

Hence, we conducted a real-world comparison of the two regimens in patients with PNETs treated with either PRRT or CAPTEM at our center. Primarily, we aimed to compare PFS and ORR in patients who received PRRT or CAPTEM for metastatic PNETs in a disease setting where they had received neither of the two regimens. Furthermore, we performed subgroup analyses looking at variables affecting PFS for each of these systemic regimens.

## 2. Materials and Methods

### 2.1. Subject Selection and Outcomes Evaluation

Patients with metastatic well-differentiated PNETs seen at our center between September 2013 and September 2023 were included. Poorly differentiated neuroendocrine carcinomas (NECs) were excluded from this study. The distinction between NETs and NECs was based on pathologic diagnoses on file in patients’ electronic medical records. These diagnoses at our center are reported based on the 2017 (and subsequently 2022) World Health Organization (WHO) classification system for pancreatic neuroendocrine neoplasms, which defines well-differentiated grade 3 neuroendocrine tumors (grade 3 NETs) [17,18]. Electronic health records were reviewed, and clinicopathological data were abstracted. For cases with tumor next-generation sequencing (NGS) results available from CLIA-certified platforms, *MEN1*/*DAXX*/*ATRX* mutational status data were obtained. The review of records was approved by the Institutional Review Board at the University of Chicago and was in accordance with the Belmont Report for retrospective review of records and waiver of consent.

Patients who received at least 2 cycles of PRRT or 3 cycles of CAPTEM for metastatic PNETs were included. Patients without the availability of follow-up imaging results at least six months after the start of therapy were excluded. Response to therapy was assessed based on Response Evaluation Criteria in Solid Tumors (RECIST) v1.1 [19]. The primary outcomes analyzed were PFS and ORR. PFS was calculated as the time from cycle 1, day 1 of treatment to the date of documented disease progression per RECIST v1.1, or death, whichever occurred first. Patients whose disease had not progressed at the time of review were censored at the date of their last available stable imaging. For cases that were not evaluable per RECIST v1.1, the date of clinical progression was determined by the treating physician based on imaging findings and radiologists’ interpretation. ORR was recorded following a review of computed tomography (CT) and/or magnetic resonance imaging (MRI) findings. ORR was defined as the proportion of patients who achieved complete response (CR) or partial response (PR) at any point during their follow-up after cycle 1, day 1 of treatment, as per RECIST v1.1.

For analyzing the primary outcomes of PFS and ORR, if patients were sequentially treated with both regimens, the outcomes after the initial treatment received out of the two were considered. Hence, primary PFS (PFS) and primary ORR (ORR), when described in regard to PRRT, denote the PFS and ORR, respectively, in patients who were treated with PRRT either alone or in a line of treatment prior to receiving CAPTEM. Similarly, PFS and ORR in relation to CAPTEM refer to outcomes in patients who received CAPTEM either alone or in a line of treatment before PRRT during their treatment course.

Secondary outcomes were time to response (TTR) and overall survival (OS) in patients who received both PRRT and CAPTEM. Among patients who achieved CR/PR, TTR was defined as the duration from cycle 1, day 1 of treatment to the date of first imaging showing objective response as per RECIST v1.1.

A subsequent subgroup analysis for PFS1, PFS2, ORR1, and ORR2 was conducted in patients who were sequentially treated with both regimens, with the subsequent regimen being administered only after progression on the initially received regimen. PFS1 and ORR1 refer to the PFS and ORR, respectively, with the initial regimen administered out of PRRT and CAPTEM in patients who received both. PFS2 and ORR2 refer to the PFS and ORR, respectively, for the subsequent regimen out of PRRT or CAPTEM received by these patients. These treatment groups will henceforth be referred to as PRRT after initial CAPTEM and CAPTEM after initial PRRT, respectively. The division of study subjects into subgroups for analysis is further described in the Section 3.

OS was calculated from cycle 1, day 1 of treatment to the date of death or last known clinical contact. Patients who had less than one year of follow-up after the subsequent regimen out of PRRT or CAPTEM received were excluded from the OS analysis. Median follow-up was calculated from the date of cycle 1, day 1 of treatment to the date of last known clinical contact or death.

### 2.2. Treatment Protocols

The PRRT treatment protocol included four cycles of ^177^Lu-DOTATATE at 200 millicuries per dose and was administered at 8-week intervals. Concurrent somatostatin analog (SSA) therapy was allowed and was withheld for at least 28 days before PRRT and restarted within 24 h of PRRT treatment.

The CAPTEM treatment protocol consisted of capecitabine 750 mg/m^2^ twice a day for days 1–14 and temozolomide 200 mg/m^2^ once daily on days 10–14, both given in 28-day cycles. The duration of treatment was at the discretion of the treating physician.

### 2.3. Statistical Analysis

Statistical analyses were performed using BlueSky Statistics (version 10.3.2). Continuous variables are reported as medians and interquartile ranges (IQR) and compared using the Wilcoxon test for independent samples. Categorical variables are reported as proportions in each group and compared using the Chi-Square test or Mid-P exact test, as applicable (compared using OpenEpi version 3.01). Survival analyses were conducted using the Kaplan–Meier estimator and Cox proportional hazards regression models. Univariable Cox proportional hazards regression modeling was used to analyze the risk of progression in subgroups of PNETs. Time-to-response comparison was performed using the log-rank test. The threshold for statistical significance for all tests was *p* < 0.05.

## 3. Results

### 3.1. Demographic and Clinicopathologic Features

**Figure 1:**
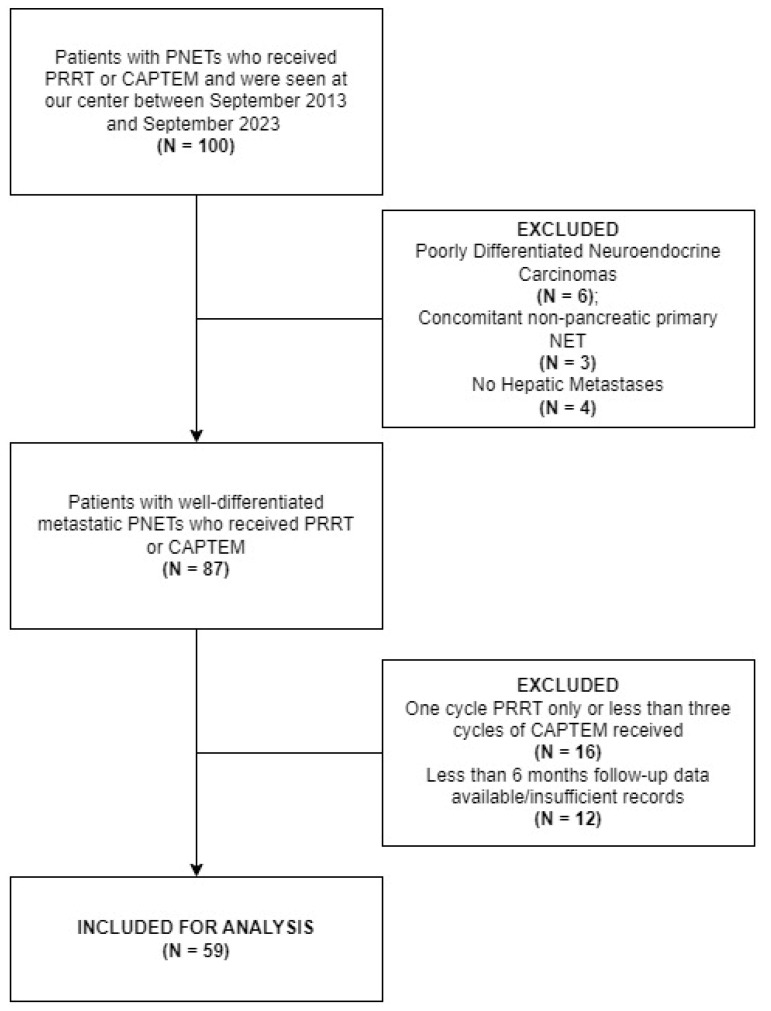
STROBE flowchart for subject selection. PNETs = pancreatic neuroendocrine tumors; PRRT = Peptide Receptor Radionuclide Therapy; CAPTEM = Capecitabine/Temozolomide; NET = neuroendocrine tumor.

Fifty-nine patients with metastatic well-differentiated PNETs who received either PRRT or CAPTEM after receiving at least one prior line of systemic therapy were included in the final analysis. The subject selection process is described in Figure 1. Median follow-up across the cohort after cycle 1, day 1 of PRRT or CAPTEM was 31.7 months (IQR, 24.68 months–47.92 months). Tumor NGS results were available in 31 patients (52.54%). The median age at the time of initial treatment (with either PRRT or CAPTEM) in the cohort was 57.48 years (IQR, 43.75–63.40). Thirty patients (50.84%) were assigned male sex at birth, while 29 patients (49.15%) were female. In 54 patients with tumor grades recorded, the grade was 1 or 2 in 41 patients (75.93%), while 13 patients (24.07%) had grade 3 disease. Seventeen patients (28.81%) received systemic therapy other than SSA (everolimus/sunitinib/other cytotoxic chemotherapy/immunotherapy) prior to receiving PRRT or CAPTEM. Fifteen patients (25.42%) underwent surgical intervention for metastatic disease, which included debulking of liver metastases with or without primary tumor removal prior to receiving PRRT or CAPTEM. A comparison of the two groups is presented in Table 1.

**Table 1:** Clinicopathological characteristics of the study cohort stratified by initial/only treatment.

	CAPTEM		PRRT		*p*-Value
	n	% of available	n	% of available	
**Total**	30		29		
**Median Age at C1D1 (IQR)**	49.34 (40.48–58.17)		60.33 (55.79–66.05)		0.02 *
**SAAB**					0.7
Male	14	46.67%	15	51.72%	
Female	16	53.33%	14	48.28%	
**Grade (N = 54)**	27		27		0.34
1 or 2	19	70.37%	22	81.48%	
3	8	29.63%	5	18.52%	
**ECOG (N = 58)**	29		29		0.42
0	16	55.17%	19	65.52%	
1 or 2	13	44.83%	10	34.49%	
**Extrahepatic Metastases**	19	63.33%	20	68.97%	0.65
**Bone Metastases**	9	30%	11	37.93%	0.52
**Baseline Chromogranin A Levels > 2 ULN (N = 50)**	13/21	61.90%	16/29	55.17%	0.63
**Median Ki-67 Index (IQR; N = 52)**	13.30 (5.00–21.00)		7.90 (3.50–18.50)		0.13
***MEN1*/*DAXX*/*ATRX* Mutation (N = 31)**	9/17	52.94%	10/14	71.43%	0.29
**Prior Surgical Intervention for Metastatic Disease**	1	3.33%	14	48.28%	<0.001 *
**Prior Systemic Therapy other than SSA**	8	26.67%	9	31.03%	0.71

**Figure 2:**
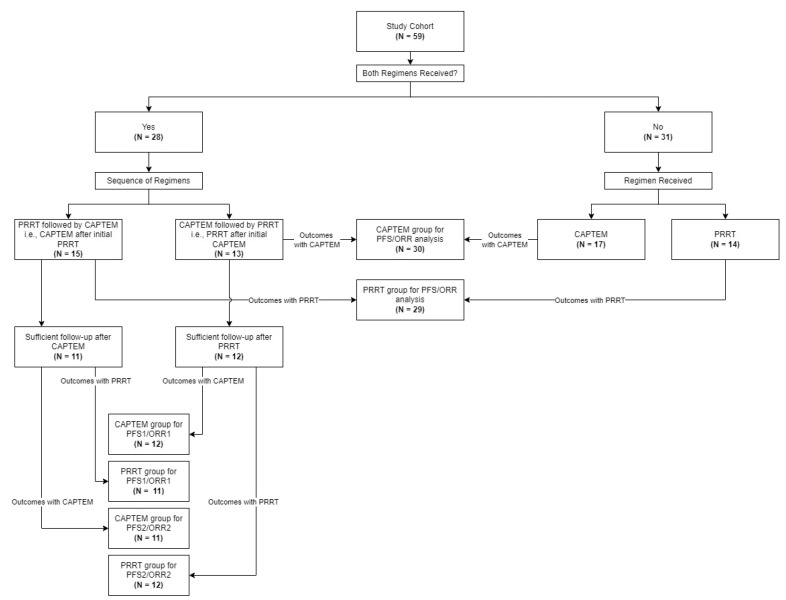
Flowchart for subject grouping for analyses. PRRT = Peptide Receptor Radionuclide Therapy; CAPTEM = Capecitabine/Temozolomide; PFS = progression-free survival; ORR = objective response rate.

PRRT = Peptide Receptor Radionuclide Therapy; CAPTEM = Capecitabine/Temozolomide; C1D1 = cycle 1, day 1 of initial/only treatment out of PRRT and CAPTEM; SAAB = Sex Assigned At Birth; ECOG = Eastern Cooperative Oncology Group; ULN = upper limit of normal; SSA = somatostatin analog; * = indicates a statistically significant difference; NB: PRRT group consists of patients who received PRRT alone or received it in a line of treatment prior to receiving CAPTEM; CAPTEM group consists of patients who received CAPTEM alone or received it in a line of treatment prior to receiving PRRT. See Figure 2 for more details.

### 3.2. Treatments Received and Group Comparison

The grouping of patients for analysis is described in Figure 2. Twenty-nine patients (49.15%) were considered in the PRRT treatment group for PFS and ORR analysis. Twenty-four of twenty-nine patients (82.76%) received the full course of four planned cycles of PRRT. One patient (3.45%) had their treatment terminated after three cycles, and four patients (13.79%) were able to receive treatment for only two out of the four planned cycles. Thirty patients received CAPTEM with regard to PFS and ORR analysis. Treatment was administered in 28-day cycles. The median number of cycles (or months of treatment) received was 12 (range, 3–44; IQR, 7–12). A comparison of baseline clinicopathological features between the PRRT and CAPTEM treatment groups, stratified based on the initial/only treatment received, is presented in Table 1. The median age at treatment was higher in the PRRT group (60.33 years vs. 49.34 years; *p* = 0.02). More patients underwent surgical intervention for metastatic disease prior to treatment in the PRRT group (1/30 vs. 14/29; *p* < 0.001). The groups did not vary significantly at baseline for any other characteristics.

### 3.3. Primary Outcomes Evaluation–Entire Cohort

**Figure 3:**
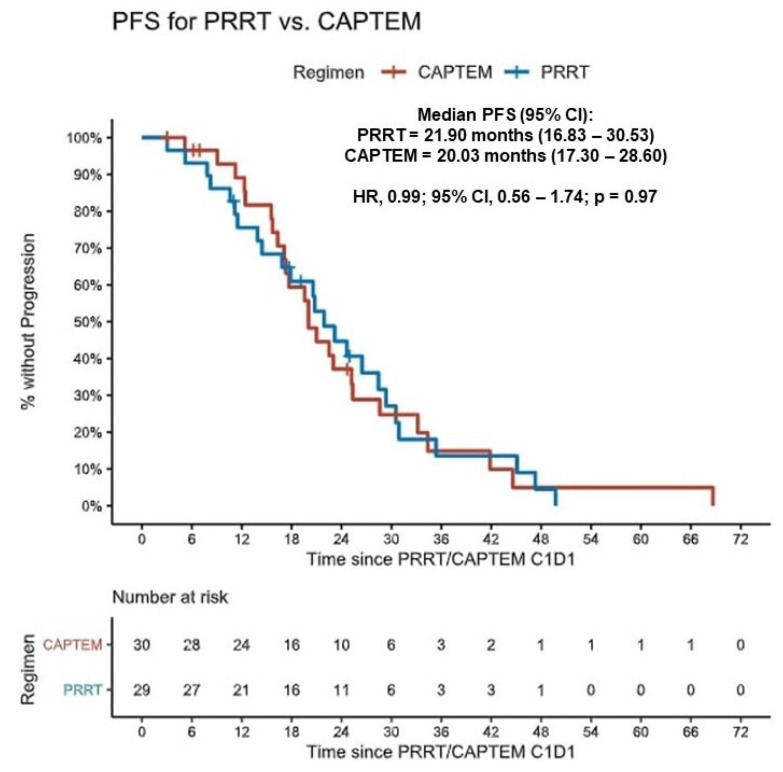
Kaplan–Meier estimates of progression-free survival according to the treatment received. PFS = progression-free survival; PRRT = Peptide Receptor Radionuclide Therapy; CAPTEM = Capecitabine/Temozolomide; C1D1 = cycle 1, day 1; NB: Primary PFS is depicted in this figure.

All patients in the cohort were considered for primary PFS analysis as described in the methodology. There was no significant difference in PFS across the whole cohort between the two treatment groups (PRRT = 21.90 months vs. CAPTEM = 20.03 months; HR 0.99; 95% CI, 0.56–1.74; *p* = 0.97). A Kaplan–Meier estimator for PFS is shown in Figure 3.

**Figure 4:**
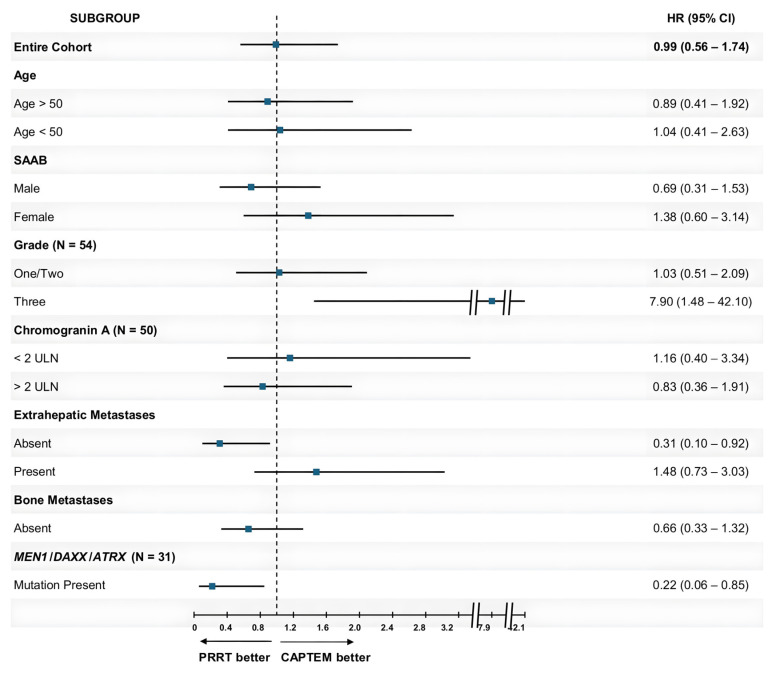
Subgroup analyses for progression-free survival across the cohort. HR = Hazard Ratio; CI = Confidence Interval; SAAB = Sex Assigned At Birth; ULN = upper limit of normal; PRRT = Peptide Receptor Radionuclide Therapy; CAPTEM = Capecitabine/Temozolomide. NB: Hazard Ratios presented are based on univariable Cox proportional hazards regression analysis; HRs from the bone metastases present in the subgroup and *MEN1*/*DAXX*/*ATRX* wild-type subgroup were omitted from this figure for ease of visualization.

Subgroup analyses for PFS were subsequently performed for baseline clinicopathological features and the presence of *MEN1*, *DAXX*, and/or *ATRX* mutations (seen in 19 out of 31 patients with tumor NGS available, 61.29%). PRRT showed significantly lower risk of progression as compared to CAPTEM in patients without extrahepatic metastases at presentation (n = 20; HR 0.31; 95% CI, 0.10–0.92; *p* = 0.03) and in patients who had *MEN1*, *DAXX*, and/or *ATRX* mutations (n = 19; HR 0.22; 95% CI, 0.06–0.85; *p* = 0.03). PRRT showed a higher risk of progression in patients with grade 3 disease (n = 13; HR 7.90; 95% CI, 1.48–42.10; *p* = 0.02). PRRT trended toward worse PFS in patients with bone metastases (n = 20; HR 2.84; 95% CI, 0.85–9.52; *p* = 0.09). The detailed results of the subgroup analysis are shown in Table 2. A visual overview of these results is presented in Figure 4.

**Figure 5:**
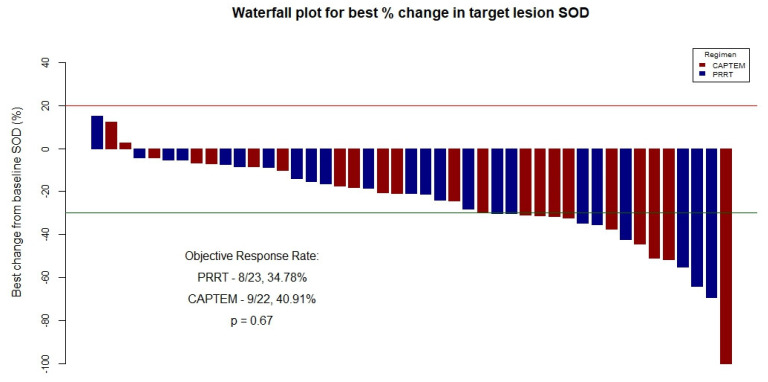
Best objective response as per RECIST v1.1 seen in all evaluable (n = 45) cases based on maximal percentage of tumor reduction from baseline sums of diameters of target lesions and association with treatment received. SOD = Sum Of Diameters; CAPTEM = Capecitabine/Temozolomide; PRRT = Peptide Receptor Radionuclide Therapy. NB: Red line indicates the cutoff for progressive disease (PD), i.e., +20%; blue line indicates the cutoff for partial response (PR), i.e., −30%. Primary ORR is depicted in this figure.

With regard to ORR analysis, 45 patients (PRRT = 23; CAPTEM = 22) had appropriate imaging results available for objective response evaluation as per RECIST v1.1. In the PRRT group, eight patients had a partial response (PR) as their best response and fifteen patients had stable disease (SD). In the CAPTEM group, one patient had complete response (CR), eight patients had PR, and the remaining thirteen patients had SD. There was no significant difference in ORR between the two groups (PRRT, 8/23, 34.78%, vs. CAPTEM, 9/22, 40.91%; *p* = 0.67). A visual overview of ORR across the cohort is presented in Figure 5.

CAPTEM (n = 9) responders showed a shorter time to response than PRRT (n = 8) responders (6.03 months vs. 11.15 months; *p* = 0.03). Notably, when considering both initial and subsequent treatments (see Section 2.1), seven patients (23.33%) were able to undergo surgical debulking owing to satisfactory disease control after receiving CAPTEM, while only one patient underwent surgical debulking (3.45%) after receiving PRRT.

### 3.4. PFS1, PFS2, ORR1, and ORR2 Analysis—Cohort with Both Treatments Received

A total of 28 patients (CAPTEM after initial PRRT = 15; PRRT after initial CAPTEM = 13) in the study cohort had received both treatments at the time of review of records. Twenty-three out of twenty-eight patients had sufficient follow-up data as described previously for PFS2 analysis. A total of 11/23 (47.83%) patients received PRRT followed by CAPTEM (CAPTEM after initial PRRT). A total of 12/23 (52.17%) patients received CAPTEM followed by PRRT (PRRT after initial CAPTEM). Five patients received at least one other line of treatment (systemic therapy, stereotactic body radiation therapy, and/or liver-directed therapy) between PRRT and CAPTEM. No patients underwent surgical debulking between PRRT and CAPTEM treatments. Eleven patients received at least one other treatment after receiving both PRRT and CAPTEM (systemic therapy, stereotactic body radiation therapy, surgical debulking, liver transplant, and/or liver-directed therapy). The groups, when stratified based on the initial treatment received out of the two (CAPTEM after initial PRRT vs. PRRT after initial CAPTEM), did not vary significantly in terms of age (58.96 years vs. 44.36 years; *p* = 0.45), the proportion with grade 3 disease (N = 22; 2/11 vs. 3/11; *p* = 0.66), presence of extrahepatic metastases (6/11 vs. 9/12; *p* = 0.35), baseline chromogranin A elevation > 2 times upper limit of normal (N = 18; 5/11 vs. 5/7; *p* = 0.33), systemic therapy other than SSA prior to treatment (3/11 vs. 3/12; *p* = 0.91), or surgical interventions for metastatic disease prior to treatment (5/11 vs. 1/12; *p* = 0.06).

Twenty-three patients were eligible for PFS1 and PFS2 comparison as described previously. There was no significant difference in PFS1 between the two treatment groups (initial PRRT = 20.57 months vs. initial CAPTEM = 17.30 months; HR, 1.69; 95% CI, 0.67–4.29; *p* = 0.27). Eighteen patients out of these twenty-three (initial PRRT = 10; initial CAPTEM = 8) had appropriate imaging data for ORR1 analysis. In the group that received initial PRRT, four patients had PR as their best response, and six patients had SD. In the group that received initial CAPTEM, two patients showed PR, and six patients showed SD. ORR1 did not vary significantly between the two groups (initial PRRT, 4/10, 40%; vs. initial CAPTEM, 2/8, 25%; *p* = 0.56).

PFS2 did not vary significantly between the two groups (CAPTEM after initial PRRT = 9.57 months vs. PRRT after initial CAPTEM = 12.52 months; HR, 1.66; 95% CI, 0.66–4.15; *p* = 0.28). With regard to ORR2 analysis, 17 patients (CAPTEM after initial PRRT = 8; PRRT after initial CAPTEM = 9) had appropriate imaging results available. In the group that received CAPTEM after initial PRRT, three patients had PR as their best response, four patients had SD, and one patient had PD. In the group that received PRRT after initial CAPTEM, two patients showed PR, six patients showed SD, and one patient showed PD. ORR2 did not vary significantly between the two groups (CAPTEM after initial PRRT, 3/8, 37.5%; vs. PRRT after initial CAPTEM, 2/9, 22.22%; *p* = 0.55). An overview of these results is presented in Table 3.

Of note, there was no significant difference in PFS in patients who had not received both treatments (N = 31; PRRT = 14, CAPTEM = 17) at the time of review of records (PRRT = 21.90 months vs. CAPTEM = 20.00 months; HR 0.87; 95% CI, 0.36–2.13; *p* = 0.77).

### 3.5. Overall Survival Analysis

An analysis of overall survival from cycle 1, day 1 of the first treatment received was conducted for patients who received both PRRT and CAPTEM in any sequence. Twenty-three patients (38.98%) met the inclusion criteria described for OS analysis. OS did not vary significantly based on the sequence in which PRRT (CAPTEM after initial PRRT = 11) and CAPTEM (PRRT after initial CAPTEM = 12) were received (CAPTEM after initial PRRT = 48.57 months vs. PRRT after initial CAPTEM = 50.07 months; HR, 1.20; 95% CI, 0.39–3.76; *p* = 0.75).

## 4. Discussion

Our study reports a real-world comparison of efficacy (measured in terms of PFS and ORR) in patients treated with PRRT versus CAPTEM after progression on SSAs for metastatic well-differentiated PNETs. The objective of this study was to compare the efficacy of these regimens to uncover trends in outcomes that may inform treatment sequencing. 

Our reported PFS for PNETs treated with PRRT of 21.90 months aligns with data reported from large PPRT registries by Aalbersberg et al. (23 months in patients treated with ^90^Y PRRT/^177^Lu PRRT or both) and Mitjavila et al. (19.80 months with ^177^Lu-DOTATATE) [12,20]. The ORR seen in our study with PRRT (34.78%) was also similar to the ORR reported by Mitjavila et al. (42.4%) [12]. Our reported PFS for CAPTEM (20.03 months) was marginally shorter than the PFS reported in the ECOG-ACRIN E2211 trial (23.2 months), although this may be explained by the fact that our data were entirely collected in a real-world setting [16]. Our ORR with CAPTEM (40.91%) was similar to the ORR reported in the ECOG-ACRIN E2211 trial (39.7%) [16].

In this study, we found that PFS was comparable between patients who received PRRT vs. CAPTEM following progression on at least one prior line of systemic therapy. While overall PFS did not differ significantly, our findings from subgroup analyses (stratification by radio-sensitizing mutations and grade in particular) highlight the need for patient-specific therapies. Patients with grade 3 disease may benefit from the utilization of CAPTEM in a line of treatment before PRRT, and patients with radio-sensitizing mutations detected (see below) may benefit from PRRT before CAPTEM. Ongoing trials (for, e.g., NCT05247905) comparing these two regimens offer an opportunity to prospectively investigate the associations reported in retrospective analyses such as ours.

The finding that more patients in the PRRT group underwent surgical debulking of liver metastases prior to treatment may be attributable to a historical bias towards the use of systemic chemotherapy in widespread metastatic disease. Notably, we found more patients undergoing debulking due to satisfactory disease control with CAPTEM as compared to PRRT (seven vs. one). The finding suggests the approach to choosing systemic therapy for patients who require tumor shrinkage and may eventually undergo surgical debulking was consistent with established patterns and recommendations [5,6].

TTR was shorter in CAPTEM responders (6.03 months) as compared to PRRT responders (11.15 months). Surgical debulking has been shown to have both palliative and survival benefits in patients with metastatic neuroendocrine tumors [21,22,23,24]. Therefore, for patients who are deemed suitable candidates for surgical debulking, CAPTEM could expedite the reduction of disease load pre-operatively. Faster TTR with CAPTEM could also lead to quicker symptomatic relief in patients who have a high disease burden.

In the limited subset of patients who received both treatments, OS did not vary significantly based on the sequence in which these regimens were utilized. PFS with the first agent utilized between the two was numerically longer in both groups (PRRT, initial 20.57 months vs. subsequent 12.52 months; CAPTEM, initial 17.30 months vs. subsequent 9.57 months). Retrospective analyses in NETs have reported a higher risk of progression in patients who receive systemic therapies prior to PRRT [12,20]. The median PFS of CAPTEM has also been reported to vary based on the line of treatment in which it is utilized for metastatic NETs [25]. While comparative analyses were limited by our sample size, our results align with these data.

Studies have also shown lower O-6-Methylguanine-DNA Methyltransferase (MGMT) expression and *MGMT* promoter methylation to be associated with improved response to temozolomide-based chemotherapy in PNETs, similar to the utility of MGMT status in glioblastoma [26,27,28]. Notably, data from the ECOG-ACRIN E2211 study has shown MGMT deficiency to be associated with greater odds of objective response with temozolomide-based therapy in PNETs [29]. Our subgroup analyses herein showed that mutations in either the *MEN1*, *DAXX*, and/or *ATRX* genes, which may radio-sensitize these tumors through their effects on DNA damage repair mechanisms, did confer a progression-free survival benefit in patients treated with PRRT [30,31,32,33]. Hence, *MGMT* status and the presence of radio-sensitizing mutations could serve as potential biomarkers that aid in choosing between PRRT and CAPTEM in clinical practice.

This study is limited by its small sample size and retrospective design. Nevertheless, our findings provide a unique real-world comparison of treatment efficacies and offer several signals for future research. Prospective validation of our observations regarding the varying effectiveness of these regimens in specific patient subgroups could greatly assist in selecting between the two in a clinical setting.

## 5. Conclusions

This real-world analysis of patients with metastatic PNETs treated with PRRT or CAPTEM showed that PFS, ORR, and OS are similar to these two therapies. PFS for patients without extrahepatic metastases and those with *MEN1*, *DAXX*, and/or *ATRX* mutations might be prolonged with PRRT. Patients with grade 3 disease might fare better with CAPTEM initially. Candidates for surgical debulking or those with tumor-induced symptoms may benefit from initial treatment with CAPTEM due to shorter TTR. Further prospective investigations of these trends to optimize treatment sequencing for PNETs are warranted.

## Figures and Tables

**Table 2:** Subgroup analysis for PFS1 comparisons between PRRT and CAPTEM.

Subgroup	PRRT Number	mPFS in Months with PRRT (95% CI)	CAPTEM Number	mPFS in Months with CAPTEM (95% CI)	HR (95% CI)	*p*-Value
Entire Cohort	29	21.90 (16.83–30.53)	30	20.03 (17.30–28.60)	0.99 (0.56–1.74)	0.97
Age						
Age > 50	22	23.17 (17.87–35.37)	14	20.03 (16.33–NR)	0.89 (0.41–1.92)	0.77
Age < 50	7	20.77 (13.90–NR)	16	21.27 (17.10–44.60)	1.04 (0.41–2.63)	0.93
SAAB						
Male	15	23.17 (20.57–NR)	14	18.43 (15.70–NR)	0.69 (0.31–1.53)	0.36
Female	14	16.83 (11.5–NR)	16	23.00 (17.67–NR)	1.38 (0.60–3.14)	0.76
Grade (N = 54)						
One/Two	22	26.47 (20.77–35.37)	19	23.00 (20.03–NR)	1.03 (0.51–2.09)	0.92
Three	5	7.83 (5.20–NR)	8	16.33 (11.23–NR)	7.9 (1.48–42.10)	0.02
Baseline Chromogranin A Levels (N = 50)						
<2 ULN	13	26.47 (11.50–NR)	8	20.03 (17.67–NR)	1.16 (0.40–3.34)	0.79
>2 ULN	16	21.90 (14.43–NR)	13	20.03 (16.33–NR)	0.83 (0.36–1.91)	0.65
Extrahepatic Metastases						
Absent	9	26.47 (20.77–NR)	11	17.67 (15.70–NR)	0.31 (0.10–0.92)	0.03
Present	20	20.57 (14.43–NR)	19	25.20 (17.30–44.60)	1.48 (0.73–3.03)	0.28
Bone Metastases						
Absent	18	24.63 (20.77–45.10)	21	20.03 (17.30–52.33)	0.66 (0.33–1.32)	0.24
Present	11	17.87 (10.60–NR)	9	28.60 (16.33–NR)	2.84 (0.85–9.52)	0.09
*MEN1*/*DAXX*/*ATRX* Mutation (N = 31)						
Absent	4	18.80 (7.83–NR)	8	24.08 (12.33–NR)	2.4 (0.53–10.87)	0.26
Present	10	28.43 (17.87–NR)	9	18.67 (15.53–NR)	0.22 (0.06–0.85)	0.03

PRRT = Peptide Receptor Radionuclide Therapy; mPFS = median progression-free survival; CI = Confidence Interval; CAPTEM = Capecitabine/Temozolomide; HR = Hazard Ratio; SAAB = Sex Assigned At Birth; ULN = upper limit of normal. NB: Hazard Ratios and *p*-values presented are based on univariable Cox proportional hazards regression analysis; NB: PRRT group consists of patients who received PRRT alone or received it in a line of treatment prior to receiving CAPTEM; CAPTEM group consists of patients who received CAPTEM alone or received it in a line of treatment prior to receiving PRRT.

**Table 3:** PFS and ORR comparisons in patients who were sequentially treated with both regimens and had sufficient follow-up (N = 23).

Outcomes	PRRT	CAPTEM
Objective Response Rate		
ORR1 (N = 18)	4/10, 40%	2/8, 25%
*p*-value	0.56	
ORR2 (N = 17)	2/9, 22.22%	3/8, 37.5%
*p*-value	0.55	
Progression-Free Survival		
PFS1 [mPFS (95% CI)]	20.57 (13.90–NR)	17.30 (15.53–NR)
*p*-value	0.27	
PFS2 [mPFS (95% CI)]	12.52 (8.17–NR)	9.57 (8.63–NR)
*p*-value	0.28	

PRRT = Peptide Receptor Radionuclide Therapy; CAPTEM = Capecitabine/Temozolomide; ORR = objective response rate; PFS = progression-free survival; mPFS = median progression-free survival; CI = Confidence Interval; NR = Not Reached. NB: Please refer to methods, results, and Figure 2 for interpretation of PFS1/PFS2 and ORR1/ORR2; *p*-values for PFS comparisons are reported from univariable Cox proportional hazards regression analysis.

## Data Availability

The raw data supporting the conclusions of this article will be made available by the authors upon request.
